# Supplementary material for: A scientometric analysis of fairness in health AI literature
Source: PLOS Glob Public Health. 2024 Jan 19;4(1):e0002513. doi: 10.1371/journal.pgph.0002513 (PMC10798451; doi:10.1371/journal.pgph.0002513)
Supplement: S1 Appendix — (PDF) [file pgph.0002513.s001.pdf]

APPENDIX

Acknowledgements

- OpenStreetMap
  - Data available under Open Database License
- Go-cart.io
  - Gastner MT, Seguy V, More P. Fast flow-based algorithm for creating density-equalizing map projections. Proc Natl Acad Sci USA 115(10):E2156–E2164 (2018)
  - Images generated by go-cart.io can be distributed under a permissive license, CC-BY.

Code:
Data and code repository can be found at https://github.com/anpetushkov/fairness-community

Fig A: Citation OLS Results.

Variable	Coefficient	Standard Error	t	P>|t|	[0.025 0.975]
% male	-5.2626	13.841	-0.380	0.704	[-32.526 22.001]
% White	-5.4907	18.604	-0.295	0.768	[-42.136 31.155]
First author (white)	-0.9839	10.761	-0.091	0.927	[-22.179 20.211]
First author (male)	4.2611	7.728	0.551	0.582	[-10.961 19.483]
Last author (white)	-6.2623	10.376	-0.604	0.547	[-26.700 14.175]
Last author (male)	8.6737	8.116	1.069	0.286	[-7.313 24.660]
# of authors	2.3170	0.691	3.354	0.001	[0.956 3.678]
Year	3.7675	0.833	4.520	0.000	[2.126 5.409]

Fig B: Citation Logit Regression Results

Variable	Coefficient	Standard Error	z	P>|z|	[0.025 0.975]
% male	-0.2201	0.572	-0.385	0.700	[-1.340 0.900]
% White	0.3402	0.760	0.447	0.655	[-1.150 1.831]
First author (white)	0.1778	0.440	0.405	0.686	[-0.684 1.039]
First author (male)	0.2559	0.317	0.808	0.419	[-0.365 0.877]
Last author (white)	0.1884	0.423	0.446	0.656	[-0.640 1.017]

Last author (male)	-0.3192	0.333	-0.958	0.338	[-0.972 0.334]
# of authors	0.0030	0.028	0.105	0.916	[-0.052 0.058]
Year	-0.0686	0.039	-1.780	0.075	[-0.144 0.007]

Fig C: Funding Breakdown by demographic for First Authors

	First author			
	White	Non-White	Male	Female
# of papers	150	103	145	108
% Funded	.533	.437	.503	.481
P-value	0.133		.731	

Fig D: Funding Breakdown by demographic for Last Authors

	Last author			
	White	Non-White	Male	Female
# of papers	156	97	173	80
% Funded	.532	0.433	.468	.550
P-value	.126		.228	

Maps on Tableau:
https://public.tableau.com/app/profile/tiffany.chua/viz/AIintheHealthcareFairnessCommunity/SpatialDash
https://public.tableau.com/app/profile/jonathan6077/viz/IstheFairnessCommuntyFair/IstheFairnessCommunutyFair?publish=yes&fbclid=IwAR2yPmckXCEQnzHyDdt6L7brIAkCP9zOEprcHQb9anYmrW4NTOnHjDzUUas

Search String:

("Artificial Intelligence"[mesh] OR "Pattern Recognition, Automated"[mesh] OR "Data Mining"[Mesh] OR artificial intelligence[tiab] OR computational intelligence[tiab] OR machine intelligence[tiab] OR intelligent automation[tiab] OR intelligent system*[tiab] OR machine learning[tiab] OR deep learning[tiab] OR deep network*[tiab] OR supervised learning[tiab] OR natural language process*[tiab] OR neural net*[tiab] OR perceptron*[tiab] OR algorithmic decision making[tiab] OR predictive care tool*[tiab] OR predictive medicin[tiab] OR predictive model*[tiab] OR data mining[tiab]) AND ("Health Equity"[mesh] OR "Social Discrimination"[Mesh] OR "Healthcare Disparities"[mesh] OR fairness[tiab] OR egalitarian*[tiab] OR distributive justice[tiab] OR ((inequalit*[tiab] OR disparit*[tiab] OR inequit*[tiab] OR equity[tiab] OR equality[tiab] OR underrepresent*[tiab]) AND (health*[tiab] OR healthcare[tiab] OR racial[tiab] OR

ethnic[tiab] OR sex[tiab] OR sexual[tiab] OR socioeconomic[tiab] OR economic[tiab]))) **mesh terms removed for the Google Scholar searches.

Table A - Distribution of authors across Gender and Race

	**First author**	**Last Author**	Other Authors	Total
**Gender**				
Male	220 (58.8%)	243 (68.8%)	727	1190
Female	155 (41.3%)	110 (31.2%)	529	794
**Race**				
White	234 (62.4%)	234 (66.3%)	802	1270
Non - White	141 (37.6%)	119 (33.7%)	436	696
Hispanic	*14 (3.7%)*	*11 (3.1%)*	49	74
Asian	*103 (27.5%)*	*96 (27.2%)*	334	533
Black	*24 (6.4%)*	*12 (3.4%)*	53	89
