# Supplementary material for: A scientometric analysis of fairness in health AI literature
Source: PLOS Glob Public Health. 2024 Jan 19;4(1):e0002513. doi: 10.1371/journal.pgph.0002513 (PMC10798451; doi:10.1371/journal.pgph.0002513)
Supplement: Attachment — Submitted filename: Response to Reviewers - AI Fairness.pdf [file pgph.0002513.s003.pdf]

To the Editor-in-Chief,
PLOS Global Public Health

Re: Manuscript PGPH-D-23-00817
Title: “A scientometric analysis of fairness in health AI literature”

Dear Editors and Reviewers,

I hope this message finds you well. Thank you for your valuable feedback and constructive comments on our manuscript. We appreciate the time and effort put into the review process and value the opportunity to improve our work. We have made revisions as suggested and are grateful for your time and critical insight, which has improved the study further.

Below, we address each of the points made.

Editor Comments

1. References
    a. Reference list was updated as a result of the revisions made. List is now complete and correct.
2. Figure Files
    a. Separate figure files have been added
3. Supporting Information
    a. Supporting Information Files uploaded
4. Copyright Issues
    a. Citations added

Reviewer #1 Revisions

ABSTRACT
1. Regarding authors, it looks like 22 authors wrote individually (i.e., first author only). I am curious to know the specific demographics/information about these 22 individuals and if there is any difference.

- Demographic information about the authors of the paper are listed before the authors are listed in the manuscript. It is also seen at lines 150-154.

2. Why are the last authors singled out? I need more context on this and why this is relevant (I understand first author).

- Sentences added at Line 145 and Line 289 discussing the role of last authors

INTRODUCTION

3. The concept of “trustworthy AI” is introduced in this paragraph but is not defined. Can you expand on this concept and clearly define it? (Line 51)

- Lines 43-49 now expand on the concept of Trustworthy AI and clearly define it.

4. When did the ProPublica article come out that caused the increased momentum in fairness in health AI? (Line 51)
    - Date added. “In 2016,” (Line 52)

5. I’m trying to better understand the connection between health AI and the ProPublica article about judicial courts. Is it specifically health AI that gained momentum or the AI momentum in general? I would like to see the idea of health AI and the effects of AI in healthcare more explicitly discussed. (Line 54)
    - Added Line 55-59 to expand on this.

6. Overall, I see the paper with three main concepts: artificial intelligence, trustworthy AI & fairness, and health AI literature. I would like to have more background information on these three.
    - We believe the added revisions help clarify this point.

7. I am curious about other similar bibliometric studies. How does this compare with other fields? Other studies?
    - We believe this to be outside the scope of this specific study but is an avenue worth investigating.

METHODOLOGY

8. How did you choose the three journals PubMed, Google Scholar, and Embasse? Also, a small detail but each time they are listed they are in different order -- to be consistent, could you maybe choose one order? (e.g. Lines 96 and 79).
    - Ordering of databases made more consistent. Line 81 explains why these databases were chosen.

DISCUSSION

9. I appreciate that the discussion highlights a synthesis of the findings and then lays out the practical implications and applications of your research. To further structure your paper, a suggestion could be to separate the discussion into discussion and then implications/actions.
    - Possible Actions heading added.

10. I also believe some of the possible actions could even be further expanded on. For example, the first action is to support research capacity development in lower-income and lower-middle-income countries. Can you be a bit more specific with how you can support this? (Line 368).
    - Expanded on how to support research capacity development in lower-income and lower-middle-income countries (Line 333-347) and at other points when possible.

11. I like how you state that there is a gender and ethnicity disparity in critical care medicine (Line 345). Since your analysis is on fairness in AI within the healthcare field, can you elaborate and expand on how your findings compare within that field?
    - Expanded on this in Lines 292 - 304 to better reflect this.

12. Thank you also for discussing the limitations regarding both article selection and categorizing authors’ identities.

CONCLUSION

13. You summarize your paper nicely and succinctly, noting the importance of equity and inclusivity for future research as well as the importance of the AI research community at large.
14. My only other note here is that the second sentence could be re-worked as it runs on: there are several ideas floating around in this sentence and I find it hard to follow along. (Lines 447-450)
- Reworked conclusion for clarity.

Reviewer #2 Revisions

ABSTRACT
1. Included papers. 375, were repeated in the methods and results. I suggest this is deleted from the methods in the abstract.
- This was deleted as suggested.
2. Authors should be consistent with the use of decimal place, I suggest 1 decimal place is used as it is largely used in the manuscript.
- Decimal places were standardized throughout the document as suggested.
3. "The linear and logistic regressions were analyzed using Python"........ what was this analysis for?
- “The linear and logistic regressions to evaluate factors that influence the numbers of citations and presence of funding were analyzed using Python” (Line 10)
4. The sentence before the last paragraph in their conclusion seems more like a finding than a conclusion. Authors can consider and revise.
- Conclusion in the abstract section was revised.
5. "Most authors were from US, Canada and United Kingdom"....Can authors quantify what is referred to as "Most".
- Line 22 was edited for clarity - “The top three countries with the most authors were the USA (986; 49.7%), Canada (142; 7.2%), and the United Kingdom (117; 5.9%)”

INTRODUCTION

6. Line 51 -54 may need revision. Long sentence and not clear.
- Lines 51 - 54 were changed to two sentences for clarity.
7. It will of interest if authors expand on the fairness of AI in the introduction
- Line 53-54: Definition from European Commission added.

METHODOLOGY
8. When was the literature search conducted? (period of search?)
- Line 80 addresses this. “Literature search was conducted in June 2022
9. How was papers excluded apart from the inclusion criteria, At what levels of were the papers screened?
- Resolved with moving Lines 190-196 (Now Lines 91-95).
10. How was the number of times a paper was cited identified? Authors should kindly elaborate
- Line 98-100 changed to clarify how paper citations were identified.

11. line 140.."authors who identified...." This aspect is not properly placed. I suggest it comes just after the inclusion criteria.

- We believe this section is best kept in its original section as it is integral to our approach of identifying author's nationality, race, and sex.

12. I suggest authors elaborate on the analysis procedure, as it stands, it does not reflect the content of the work

- Statistical analysis was reworked as seen in Tracked Changes manuscript.

13. How many research papers were included in the regression analyses?

- Line 161 now reflects the number of research papers included in the regression analysis.

14. Line 190-196 ("As scope of the analysis.....") seem wrongly placed. I suggest it is moved to the methods section.

- Moved to Methods under eligibility of articles (Now Lines 91-95).

RESULTS

15. "Regression analyses revealed that the percentage of female authors, percentage of white authors, race of authors, gender of authors, and the number of authors is not correlated with the likelihood of being cited. Publication year was the only factor affecting to be cited"
Authors should support these findings with statistical inference. Which table or figure is being referred to in the work? Similar comment in the OLS regression.

- Regression paragraphs now have proper citing of table and figures.

16. I suggest authors go through the manuscript and correct sentences starting with numbers, eg, line 333

- Sentences starting with numbers corrected throughout the document.

LIMITATION

17. Is the last paragraph part of limitations of the study? if not, authors should consider giving it appropriate heading. Other headings can also be considered in the manuscript especially the methods section

- Paper was restructured with appropriate headings.

In addition to the specific points mentioned above, we have carefully reviewed and revised the entire manuscript for clarity, grammar, and language.

We believe that these revisions significantly strengthen the quality and comprehensiveness of our manuscript. We are confident that the revised version now meets the publication criteria of PLOS Global Public Health.

Thank you once again for the opportunity to revise and resubmit our manuscript. We look forward to your feedback on the revised version.

Thank you for your continued consideration. Sincerely,

Justin Quion
Email: justinmicq@gmail.com
