# Supplementary material for: A scientometric analysis of fairness in health AI literature
Source: PLOS Glob Public Health. 2024 Jan 19;4(1):e0002513. doi: 10.1371/journal.pgph.0002513 (PMC10798451; doi:10.1371/journal.pgph.0002513)
Supplement: Attachment — Submitted filename: REVIEW_ A scientometric analysis of fairness in health AI literature_LHoemeke.docx [file pgph.0002513.s002.docx]

A scientometric analysis of fairness in health AI literature

PLOS Global Public Health

## Overall Comments & Introduction

This manuscript provides an in-depth bibliometric analysis of scholarly articles concerning the topic of artificial intelligence (AI) and medicine to further investigate the fairness of AI in literature. The authors reviewed 375 publications and reviewed corresponding multiple factors (including demographic data, funding, nationality, etc.) for each author.

With the rapid growth of AI in various fields, this article has a timely and relevant nature to it. I believe this article has the potential to contribute to the field and to PLOS Global Public Health with edits. The strength of this review lies largely in the analysis of these articles that yield significant results.

Below are more comments and feedback, divided by section, which I believe can help strengthen your manuscript. I also advise that this manuscript be re-checked and read for grammatical errors.

## Abstract

- (Maybe this is a question to you, Laura -- do you know what the abstract limitations are? It seems a bit lengthy for an abstract)
- Regarding authors, it looks like 22 authors wrote individually (i.e., first author only). I am curious to know the specific demographics/information about these 22 individuals and if there is any difference.
- Why are the last authors singled out? I need more context on this and why this is relevant (I understand first author).

## Introduction

- The concept of “**trustworthy AI**” is introduced in this paragraph but is not defined. Can you expand on this concept and clearly define it? (Line 51)
- When did the ProPublica article come out that caused the increased momentum in fairness in health AI? (Line 51)
- I’m trying to better understand the connection between health AI and the ProPublica article about judicial courts. Is it specifically health AI that gained momentum or the AI momentum in general? I would like to see the idea of health AI and the effects of AI in healthcare more explicitly discussed. (Line 54)
- Overall, I see the paper with three main concepts: **artificial intelligence**, **trustworthy AI & fairness**, and **health AI literature.** I would like to have more background information on these three.
- I am curious about other similar bibliometric studies. How does this compare with other fields? Other studies?

## Methods

- How did you choose the three journals PubMed, Google Scholar, and Embasse? Also, a small detail but each time they are listed they are in different order -- to be consistent, could you maybe choose one order? (e.g. Lines 96 and 79).

## Discussion

- I appreciate that the discussion highlights a synthesis of the findings and then lays out the practical implications and applications of your research. To further structure your paper, a suggestion could be to separate the discussion into discussion and then implications/actions.
- I also believe some of the possible actions could even be further expanded on. For example, the first action is to support research capacity development in lower-income and lower-middle-income countries. Can you be a bit more specific with how you can support this? (Line 368).
- I like how you state that there is a gender and ethnicity disparity in critical care medicine (Line 345). Since your analysis is on fairness in AI within the healthcare field, can you elaborate and expand on how your findings compare within that field?
- Thank you also for discussing the limitations regarding both article selection and categorizing authors’ identities.

## Conclusion

- You summarize your paper nicely and succinctly, noting the importance of equity and inclusivity for future research as well as the importance of the AI research community at large.
- My only other note here is that the second sentence could be re-worked as it runs on: there are several ideas floating around in this sentence and I find it hard to follow along. (Lines 447-450)
